# Effect of a school-based intervention to promote healthy lifestyles in 7–11 year old children

**DOI:** 10.1186/1479-5868-6-5

**Published:** 2009-01-21

**Authors:** Trish Gorely, Mary E Nevill, John G Morris, David J Stensel, Alan Nevill

**Affiliations:** 1 Institute of Youth Sport, School of Sport and Exercise Sciences, Loughborough University, Loughborough LE11 3TU, UK; 2 University of Wolverhampton, School of Sport, Performing Arts and Leisure, Walsall Campus, Gorway Road, Walsall, WS1 3BD, UK

## Abstract

**Background:**

Physical inactivity is recognised as a public health concern within children and interventions to increase physical activity are needed. The purpose of this research was to evaluate the effect of a school-based healthy lifestyles intervention on physical activity, fruit and vegetable consumption, body composition, knowledge, and psychological variables.

**Method:**

A non-randomised controlled study involving 8 primary schools (4 intervention, 4 control). Participants were 589 children aged 7–11 years. The intervention lasted 10 months and comprised a CD-rom learning and teaching resource for teachers; an interactive website for pupils, teachers and parents; two highlight physical activity events (1 mile school runs/walks); a local media campaign; and a summer activity wall planner and record. Primary outcome measures were objectively measured physical activity (pedometers and accelerometers) and fruit and vegetable consumption. Secondary outcomes included body mass index, waist circumference, estimated percent body fat, knowledge, psychological variables. Multi-level modelling was employed for the data analysis.

**Results:**

Relative to children in control schools, those in intervention schools significantly increased their total time in moderate-to-vigorous physical activity (MVPA) (by 9 minutes/day vs a decrease of 10 minutes/day), their time in MVPA bouts lasting at least one minute (10 minutes/day increase vs no change) and increased daily steps (3059 steps per day increase vs 1527 steps per day increase). A similar pattern of results was seen in a subset of the least active participants at baseline. Older participants in intervention schools showed a significant slowing in the rate of increase in estimated percent body fat, BMI, and waist circumference. There were no differences between groups in fruit and vegetable intake. Extrinsic motivation decreased more in the intervention group.

**Conclusion:**

The intervention produced positive changes in physical activity levels and body composition. It appeared to have little or no effect on consumption of fruit and vegetables. Schools are a suitable setting for the promotion of healthy lifestyles although more work, particularly focussed on dietary change, is needed in a variety of schools and social settings.

## Background

Physical inactivity is recognised as a public health issue across all ages. In children and adolescents physical activity is associated with improvements in skeletal health, CVD risk factors, adiposity, self-esteem and mental health [1]. Despite this approximately 30% of boys and 40% of girls in the UK fail to meet current physical activity guidelines of 60 minutes of moderate activity on most days of the week [2]. The increasing prevalence of overweight and obesity in young people has been attributed in part, to decreases in physical activity and increases in sedentary pursuits [3,4]. Associated with this demographic change in obesity in young people is an increased prevalence of Type 2 Diabetes [5]. If these trends are to be halted or reversed, there is an urgent need to evaluate initiatives designed to encourage healthy lifestyles in young people [6]. Public health approaches that target all children have been advocated because evidence suggests that they are more effective and are easier to implement than more selective, risk factor based approaches [7]. One way of achieving these approaches is through teaching and other activities provided to all children through schools [7,8].

Much of the work on primary school-based interventions has been conducted in the USA (e.g., [7,9-11]) and there have been calls for building of an evidence base in other countries due to concerns that cultural and educational differences make it inappropriate to simply take interventions from one country and implement them in another [12,13]. Although there are examples of primary school-based interventions in other European countries (e.g., Ireland [14], Crete [15], Germany [16] and Belgium [13]) these countries differ culturally and educationally from the UK. Within the UK itself there is limited evidence from primary school-based interventions with only two randomised controlled trials identified [8,17,18]. In the APPLES trial [17,18] the effectiveness of a whole-of-school approach to promoting physical activity and healthy lifestyles was examined among children from 10 primary schools. The programme included environmental changes (e.g., school lunches), teacher training, physical education and playground activities. No differences were observed in self-reported frequency of physical activity among children in the intervention schools compared with the control schools but there was a modest increase in vegetable consumption. The second RCT was a pilot study examining the effectiveness of lunchtime clubs in 5–7 year olds in 3 UK primary schools [8]. Participants were randomly allocated to one of 4 groups: nutrition group, physical activity group, combined group, or control group. The setting for the intervention was 25 minute long lunchtime clubs where an interactive and age-appropriate nutrition and/or physical activity curriculum was delivered over 20 weeks spread across 4 school terms. There was no clear effect of programme type on either fruit and vegetable consumption or self-reported or parent-reported physical activity with improvements generally being seen across all groups. A significant limitation of the published intervention work in the UK, and in many other studies, has been the use of self-report and parental proxy measures, of unknown reliability and validity, to assess physical activity. Such measures may not be sensitive enough to detect change [12] and in addition, they are not recommended for use with children under 10, due to cognitive limitations, rather electronic monitoring, such as accelerometry or pedometry, is advised [19]. A further limitation is that changes in overall physical activity have not always been assessed [20,21].

Based on a review of interventions to promote physical activity participation among children Salmon et al [20] concluded that interventions that incorporated school and family based components could be successful in increasing at least some elements of children's physical activity. Although the characteristics of successful primary school-based interventions are not obviously and consistently different from unsuccessful interventions [22,23] those that focus beyond just the classroom curriculum are more effective [20]. School-based components within successful interventions have included changes in physical education (either time or content), classroom based health education, activity breaks, changes to the playground, and school-sport activities. The parental component has typically involved newsletters or homework assignments to be completed with parents.

A variety of theoretical approaches have been used in the promotion of physical activity to young people, however, after conducting an intervention mapping process we concluded that successful intervention programmes, regardless of their theoretical underpinning, have consistently utilised methods that are central to Bandura's social cognitive theory (SCT) [24]. SCT emphasises modelling, rehearsal, practice, goal-setting, cueing, and reinforcement of desirable behaviours. Techniques that have been included in previous interventions based on SCT concepts include: self-monitoring and goal setting (e.g., looking at how they spend their leisure time, awarding points for being active, eating fruit and then setting targets for activity or fruit points), stimulus control (e.g., having stimuli in the home to remind them to be active), self-reinforcement (e.g., rewarding themselves when goals are reached), problem solving (e.g., looking at common barriers and ways to overcome them), and information sheets and homework sheets to parents. Small competitions on activity points have also been used.

Sport education is an instructional model designed to assist young people to become 'literate, competent and enthusiastic sports people'. A critical component of sport education approaches is a culminating or festival event at the end of the sport education 'season' [25-27]. Research has demonstrated that pupils enjoy working towards these events and find the actual days fun and enjoyable [28]. The inclusion of festival days or highlight events may therefore be a useful motivational tool, alongside individual goal setting, within school-based interventions to promote physical activity within children.

The major purpose of this non-randomised controlled field trial was to determine whether a multi-component primary school-based intervention, including classroom sessions, highlight physical activity events, and outreach to families, could improve objectively measured physical activity levels (pedometers and accelerometers) and fruit and vegetable consumption. Secondary outcomes included body composition (estimated percent body fat, BMI, waist circumference), knowledge, enjoyment, perceived competence, and motivation. The effects of the intervention were examined in the whole sample and in a sub-sample of the least active at baseline (defined as below the gender specific median of baseline steps per day). Randomisation was not possible due to the local media component of the intervention (i.e., the control schools had to come from outside the local media footprint). This paper presents the results from baseline to end of intervention (10 months).

## Methods

### Participants

Four primary schools in the north-east of England who had already agreed to take part in the "GreatFun2Run" programme were recruited for this study (540 schools in total participated in the programme). These schools were matched with 4 schools in the East Midlands of England on the basis of size, ethnicity and socioeconomic status (as reflected in the Index of Multiple Deprivation (IMD) for the school postcode. The IMD is a measure of compound social and material deprivation, calculated from a variety of data including income, employment, health, education, and housing). All participating schools were government-funded primary schools. Using an α level of 0.05 and a β level of 0.9 to detect a difference of 0.33 SD around 270 children per group were needed. This included an inflation factor for the within classroom correlation of change scores and also allowed for failure to achieve follow-up measurements in 20% of children.

**Figure 1:**
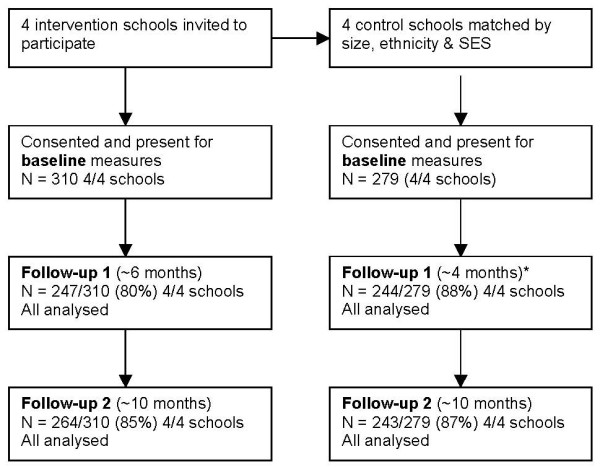
**Flow of schools and participants through study**. The difference in time to follow-up 1 between control and intervention schools was the result of the scheduling of the Christmas school holidays which meant the first data collection could not occur. These time differences are accounted for with the analysis procedures undertaken.

The characteristics of participants at baseline are described in Table 1 and the flow of schools and participants through the project is depicted in Figure 1. In total 589 children (310 intervention, 279 control) took part in the evaluation. The mean age of children at baseline was 8.8 years in the intervention schools and 8.9 years in the control schools. The majority of participants were of white British ethnicity (intervention 94.8%, control 96.5%). Despite matching schools as closely as possible on the IMD associated with the school postcode (as a broad reflection of the school catchment area) there were differences in socioeconomic status at the individual level between the two groups, with the intervention group being of lower socio-economic status than the control group when measured by the IMD for the postcode defined ward in which each participant resided. These differences were paralleled in household income with income in intervention schools being significantly lower (it is worth noting though that over 50% of parents chose not to supply this information).

**Table 1:** Baseline characteristics of intervention and control school participants.

	Intervention			Control		
	Boys	Girls	Total	Boys	Girls	Total
N	150	160	310	137	142	279
Age (years)	8.76 (.90)	8.76 (.88)	8.76 (.88)	8.89 (.88)	8.83 (.86)	8.86 (.87)
Ethnicity (white British)	96.3%	93.5%	94.8%	96.5%	96.6%	96.5%
Annual household income (£/year)	31636	30059	30886	43284	36747	39831
IMD* Low	64.2%	66.1%	65.2%	4.4%	14.7%	9.6%
Medium	26.6%	24.2%	25.3%	40.4%	41.4%	40.9%
High	9.2%	9.7%	9.4%	55.3%	44.0%	49.6%
BMI^‡^	17.58 (2.61)	18.09 (3.10)	17.84 (2.88)	17.19 (2.42)	17.46 (2.56)	17.33 (2.49)
BMI SDS^§‡^	.63 (1.08)	.58 (1.13)	.60 (1.11)	.42 (1.10)	.35 (1.00)	.39 (1.05)
Estimated % body fat^‡^	18.54 (6.36)	26.67 (5.58)	22.57 (7.23)	17.58 (6.63)	25.77 (5.60)	21.72 (7.37)
Waist circumference ^‡ ^(cm)	60.54 (6.88)	60.10 (8.88)	60.32 (7.94)	60.82 (7.55)	58.45 (6.74)	59.63 (7.24)
Steps per day^‡^	9789.34 (2929.11)	9397.79 (2559.37)	9579.42 (2735.64)	10946.47 (2947.50)	9452.42 (2654.66)	10163.49 (2888.82)
MVPA_total_^‡ ^(minutes/day)	138.85 (26.43)	113.85 (21.47)	124.72 (26.70)	125.36 (26.12)	116.56 (21.12)	120.32 (23.67)
MVPA_bout_^‡ ^(minutes/day)	52.25 (18.70)	30.73 (12.38)	40.09 (18.73)	44.73 (18.41)	30.38 (11.45)	36.51 (16.37)

Values are mean (SD) unless otherwise stated.

Notes: there was 50.1% missing data to this question; * Index of multiple deprivation – an area level measure of socioeconomic status based on the postcode of the family house. It represents a measure of compound social and material deprivation, calculated from a variety of data including income, employment, health, education, and housing; ^§ ^BMI standardised by age and gender [38]; ^‡ ^actual values at baseline unadjusted for design effects.

### Intervention

Physical education is usually taught by classroom teachers in UK primary schools and very few primary schools have specialist physical education teachers. None of the schools in the project had specialist physical education teachers. The Public Service Agreement for primary schools is for 2 hours of high quality physical education and school sport per week.

The intervention schools received the GreatFun2Run intervention which was designed and implemented by Great Run (a sports marketing and event management company). The intervention was based on Social Cognitive Theory [24]. The programme aimed to increase children's activity levels through PE lessons that taught the skills of running (via a number of sports and activities), highlight running/walking events which gave a goal to work towards, and through a range of classroom activities that reinforced children's learning and encouraged them to reflect on their activity levels and to do more voluntarily. Healthy food choices were explained and encouraged in a holistic approach to children's health education. The programme was multifaceted and comprised:

i. a CD-rom learning and teaching resource for teachers with physical education lesson plans and homework exercises plus suggestions for including health and activity related issues across the curriculum in literacy, numeracy, history, design, science, and geography lessons. The content of the CD-rom was linked to the requirements of the national curriculum and was designed by an educational specialist with expertise in distance learning in collaboration with a panel of PE and classroom staff at Keystage 2 level. The CD-rom was themed around space travel and contained 8 planets (units of work) the teachers could visit and work through (see Table 2). The CD-rom also introduced the "10 Star Rules" for good nutrition and physical activity which underpinned the programme and were the key messages for children to remember for their general health and well-being (see Table 2);

**Table 2:** Description of the 8 planets (units of work) within the intervention

Planet	Content
Activity Planet	Children were encouraged to think about current activity levels and evaluate them
Sport 2012	Introduced the Olympic games, the Olympic movement and the sports that make up the games
My Running	Introduced running for sustained periods and as an end in itself, plus famous runners, running footwear, distance, and pacing
My Safety	As being active may take children out of direct adult supervision this unit encouraged the children to think about their actions, taking care to avoid potential dangers and agreeing rules with parents/carers and road safety
Busy Bodies	Children were encouraged to think about their bodies and how they work especially in relation to aerobic exercise, it also introduced the kinds of foods we need to keep bodies healthy and provide energy to stay fit
Space Cafe	Messages on healthy eating, encouraged thinking about foods – their constituents and origins, healthy choices, fluids
Quiz Planet	Range of puzzles, games and activities to encourage the children to think about what they had learnt
Perfect Planet	Children were encouraged to think about the perfect world and what it would be like. Activities encouraged them to think of wants, needs and obligations they would have to themselves and to others to ensure they work towards this world. Planning for the future.
10 Star Rules	1. Eat breakfast every day
	2. Drink lots of fluids – don't wait until you are thirsty – especially when you are active
	3. Chase a rainbow – aim to eat a selection of different fruits and vegetables that are a variety of colours
	4. Only eat when you are hungry and stop when your stomach feels full
	5. You can eat a healthy diet that is varied and has lots of different kinds of foods in it
	6. Aim to include fish and dairy products like milk and cheese in your everyday diet to make your bones and teeth strong
	7. Be active for at least 60 minutes a day – but it doesn't have to be all in one go.
	8. Being active is good for your body and mind
	9. Take part in a variety of activities such as sport, walking, and riding your bike each week
	10. Include some running or jumping or active play each week to keep your heart and bones healthy

ii. two highlight events to give the children a goal for increasing their physical activity. These may have been participation in a 1 mile school run/walk or participation in a national campaign of 1 mile school run/walks, or participation in the local Great North Junior Run (1 mile). These events were mass participation events with the emphasis on participation not competition. The first highlight event took place in June and the second in October;

iii. an interactive website for pupils, teachers and parents to raise awareness of the need for physical activity and healthy eating. This website supported and expanded on the key health and fitness messages from the CD-rom;

iv. a local media campaign employing regional radio and print media to maintain interest and create excitement;

v. a summer activity wall planner and record.

The programme was designed to be as flexible as possible and teachers could decide when and how they used the material provided. Teachers received the intervention package in January at the start of the second school term and it was designed to be used up until the end of the school year in mid-July and then at the start of the new school year. No specific training was provided for the teachers and all instructions were contained within the pack. The children received the wall planner to plan and record their physical activity over the summer holidays. Six weeks into the new school year (October) the final 1-mile highlight event took place. Thus the intervention lasted for 10 months. Parents were engaged through homework tasks, information and publicity relating to runs, the activity planner, and by access to the web site.

The control schools continued with their usual physical education and health curriculum. The study was approved by the Ethical Advisory Committee of Loughborough University and the head teachers of participating schools. Parental consent was obtained prior to each round of data collection and parents also completed a health screening questionnaire on behalf of their child. On the day of testing all participants indicated their assent to participate and were asked to indicate that they were free of illness. A small number of children were excluded from the Multi-stage Shuttle Running Test for medical reasons (e.g., uncontrolled asthma, family history of early coronary death).

### Primary outcome measures

#### Physical activity

Daily physical activity was assessed objectively in 2 ways. All participants wore a Digiwalker SW200 pedometer for one week during waking hours. Children recorded the total number of steps taken in the previous 24 hours at the start of each school day. The steps recorded on Monday morning related to the previous 3 days and participants indicated whether they had worn the pedometer for most of Saturday and Sunday. In addition to the pedometer approximately 50% of children also wore an ActiGraph GT1M accelerometer during waking hours for this week. The ActiGraph is a uniaxial electromechanical device that records movement in the vertical plane. The sampling epoch was 5 seconds. During data processing 20 minutes of consecutive zero's was considered indicative of non-wearing and these data were deleted, minimum day length was set at 9 hours using the 70/80 rule [29], and time spent in moderate to vigorous physical activity (MVPA) was calculated using the Freedson et al [30] age specific cutpoints. Moderate activity equates to activities of 3–5.9 METS and vigorous activity to ≥ 6METS. Accelerometer data is reported in two ways: (i) total time in MVPA regardless of bout length (MVPA_total_) and (ii) total time in MVPA when only bouts of at least 1 minute duration were included (MVPA_bout_). When defining a bout an interruption of no more than 10% of epochs was allowed (i.e. within any given bout individuals could drop below the MVPA cut-off for no more than 10% of the time). In the absence of established cut points for bouts of physical activity in children [31] one minute was chosen to reflect the intermittent and short-burst nature of children's activity [32] and also be of sufficient duration to potentially elicit health or training benefits [33,34]. For both pedometers and accelerometers the first day of recording was dropped to account for likely reactivity and a minimum of 3 weekdays and 1 weekend day was required for inclusion of a participant's data in the study results.

Aerobic fitness was assessed using the Multi-stage Shuttle Running Test [35]. The test involves shuttling back and forth between cones/markers placed 20 m apart. The running pace is dictated by beeps on a CD. Each level lasts approximately 1 minute. At the end of each level the required running speed increases (i.e. the gap between beeps is reduced). Heart rate was monitored throughout the test using portable heart rate monitors (Polar Team System, Polar Electro (UK) Ltd., Warwick, UK).

### Fruit and vegetable intake

Food and beverage consumption during the previous 24 hours was assessed through one-on-one interviews with all participants. The 24 hour recall was modified from Cullen et al., [36]. Interviews worked backwards from the most recent meal and asked participants to recall what they had eaten and drunk at breakfast, lunch and evening meal. Prompts were included for between meal eating and drinking. Recalls were hand-coded for the frequency of consumption of fruit and vegetables. Due to difficulties of children accurately assessing portion size [37], participants were not asked about portion size and this is acknowledged as a limitation.

### Secondary outcome measures

#### Anthropometric measures

Height was measured (to the nearest 0.1 cm) using a stadiometer (Leicester Height Measure, seca ltd., England). Weight was measured (to the nearest 0.01 kg) using portable digital scales (seca 770, seca ltd, Birmingham, UK). From these body mass index (BMI) was calculated which was subsequently converted to age and gender specific standardised scores using the 1990 growth curves from Cole et al., [38]. Subscapular and triceps skinfold thickness was assessed using callipers (Harpenden, Baty International, England) and standard anthropometric methods. Body fat percentage was then estimated using generalised equations for prepubescent boys and girls [39]. Waist circumference was measured (to the nearest 0.1 cm) at the widest part of the torso between the xiphoid process of the sternum and the iliac.

### Knowledge of healthy lifestyles

Knowledge of healthy lifestyles was assessed using a brief (10 item) multiple choice knowledge of physical activity and nutrition test constructed for this research. The test was based on Keystage 2 National Curriculum requirements and the content of the intervention programme. A separate test was developed for each of the year groups involved because of different national curriculum requirements.

### Psychological measures

Perceived physical self-competence was assessed using a modified version of the perceived competence subscale from the Physical Self Perception Profile (PSPP-C; [40]). Enjoyment of physical activity was measured using a modified subscale from the Intrinsic Motivation Inventory [41]. Intrinsic (participating because I want to) and extrinsic (participating because I have to) motivation was assessed using two subscales from the Perceived Locus of Causality scale (PLOC; [42]). The modifications undertaken were designed to simplify the format given the age and cognitive abilities of the participants.

### Procedures

A field team of 10 researchers visited each school for a day 3 times during the evaluation period (baseline, midway, end of intervention). All measures were completed at all testing points (baseline, mid-point, and end of intervention). Participants attended two sessions of 90 minutes each on each testing day in groups of 15–30. In one session they completed the anthropometric assessments and the multi-stage shuttle test. In the other session they completed the psychological measures, the knowledge test, and the food recall interview. They were also given pedometers and accelerometers (for the 50% of the sample who wore both devices) and instructions on how and when to wear them during this session. Due to the differences in geographical location it was not possible to blind the measurement team to the intervention and control group allocation. A week after the testing a researcher returned to the school to collect the pedometers and accelerometers.

### Analysis of data

We applied a multilevel statistical model using ML-win [43] to assess changes in physical activity, fruit and vegetable consumption, body composition, knowledge and psychological variables. Multi-level modelling is an extension of ordinary multiple regression and is used where data have a hierarchy or clustered structure. That is, this approach takes into account the hierarchy among participants that exists because individuals within a class are more like each other than individuals between classes, and individuals within a school are more like each other than individuals between schools. Within the analyses schools were assumed to be a random sample at level 3, with classes a random sample at level 2, and individual children a random sample at level 1. Regression coefficients were recorded as a fixed component of our analysis, and within classes, schools and individuals, variation was only allowed at that level.

Within the results tables the β coefficient of the constant represents the baseline value for control group boys. The baseline values from the multilevel analysis represent the intercept values from the regression and are not the same as the raw data presented in Table 1. To interpret the results for the remaining components of a table an additive approach was employed. For example, to determine the baseline value for intervention boys the following equation is used: β_cons _+ β_group_. To determine the baseline value for control girls the equation is β_cons _+ β_sex_, and for intervention girls the equation is β_cons _+ β_group _+ β_sex_. The confidence intervals and p-values indicate whether a difference is significant or not.

All participants were included in the analysis regardless of how many testing sessions they actually attended. All analyses were conducted on an intention-to-treat basis. Figure 1 shows the number of participants tested at each session.

## Results

### Physical activity

Table 3 shows the results of the multi-level regression analysis for steps per day, MVPA_total_, MVPA_bout_, and distance run in the multistage shuttle run. At baseline boys in the control group took on average 11056 steps per day. Boys in the intervention group took on average 10561 steps per day. Compared to boys, girls on average took 1033 steps fewer per day, meaning that at baseline control girls took on average 10023 steps per day and intervention girls 9529 steps per. These gender differences were significant. None of the differences between intervention and control group at baseline were significant. For every month during the intervention period control participants increased their steps by 153 per day, however, intervention participants increased their steps by 316 steps per day. This difference was significant and means that at the end of the 10 month intervention participants in the intervention group were taking on average 1631 steps more per day than the controls. A similar pattern of results were observed in a subset of the least active at baseline, with control participants increasing their steps by 263 per day for every month in the intervention, but intervention participants increasing their steps by 448 steps per day for every month in the intervention (full data available upon request). This difference was significant.

**Table 3:** Multilevel regression analysis for steps/day, minutes of MVPA and distance run

	Steps/day			MVPA_total _(mins/day)			MVPA_bout _(mins/day)			Distance run (m)		
	β	CI	p	β	CI	p	β	CI	p	β	CI	p
Constant	11055.7	10244.8, 11866.6	.000	135.0	128.1, 142.5	.000	49.7	43.5, 55.8	.000	784.5	718.4, 850.7	.000
Sex	-1032.7	-1472.9, -592.5	.000	-18.0	-22.8, -13.5	.000	-19.0	-21.9, -16.0	.000	-221.0	-252.8, -189.1	.000
Group	-494.3	-1611.3, 622.6	.192	5.0	-5.2, 14.4	.181	4.6	-4.0, 13.1	.149	-8.2	-98.5, 82.0	.439
Months in intervention	152.8	82.0, 223.5	.000	-0.9	-1.7, -0.2	.005	-0.3	-0.8, 0.2	.123	3.9	-1.9, 9.7	.092
Group × month	163.2	58.3, 268.0	.001	2.0	0.7, 2.9	.000	1.3	0.5, 2.0	.000	2.3	-5.0, 9.6	.268
Age	n/a			n/a						66.2	36.5, 95.9	.000

CI = 95% confidence interval

Control participants decreased their time in MVPA_total _during the course of the intervention by almost one minute a day for every month in the intervention. In contrast, intervention participants increased their time in MVPA_total _by just over a minute a day for every month of the intervention. Therefore by the end of the intervention, participants in the intervention schools were doing 20 minutes more MVPA_total _than the control group, a significant difference of approximately 15%. There was no significant intervention effect for MVPA_total _for the subset of least active at baseline (full data available upon request). A similar pattern of results was observed within the whole sample for MVPA_bouts_, but in addition, there was a significant effect in the least active participants at baseline showing that the intervention participants increased daily MVPA_bouts _by 1.6 minutes per month, or 16 minutes per day by the end of the intervention (full data available upon request).

There were small, but non-significant, increases in the distance run during the multi stage fitness test over the intervention period, however these differences were not associated with group membership and were primarily a function of increased age.

### Fruit and vegetable intake

The results for fruit and vegetable intake can be found in Table 4. There were no significant group by time interactions nor were there any differences by gender, group, or time in intervention for fruit and vegetable intake.

**Table 4:** Multilevel regression analysis for knowledge, and fruit and vegetable intake

	Fruit and vegetable intake			Knowledge		
	
	β	CI	p	β	CI	p
Constant	2.8	3.0, 3.7	.000	5.2	4.9, 5.4	.000
Sex	0.5	-0.3, 0.2	.278	0.1	-0.1, 0.2	.239
Group	0.3	-0.6, 0.4	.401	-0.2	-0.5, 0.2	.189
Months in intervention	0.1	-0.04, .04	.480	0.1	0.06, 0.1	.000
Group × month	-0.1	-0.1, 0.1	.413	-0.03	-0.1, 0	.067

### Body composition

Table 5 shows the results of the multi-level regression analysis for estimated body fat percentage, BMI, BMI-SDS and waist circumference. Compared to boys, girls had significantly higher estimated body fat percentage (about 8% higher) and BMI (.39 BMI units, higher) and significantly lower waist circumference (about 1 cm lower) than boys. At baseline, relative to the control group, children in the intervention group had significantly higher body fat percentage (1.6% higher), BMI (1.15 BMI units higher, BMI-SDS (.44) and waist circumference (2.6 cm higher). There was a significant increase in body fat with age (about 1% per year) and the rate of increase in estimated body fat went up with age (.49), however this increase in rate was significantly lower for the intervention group (0.9% per year) compared to the control group (1.8% per year). Similar significant patterns were observed for BMI (intervention 0.4 vs. control 0.9 BMI units per year of age), BMI-SDS (intervention -.05 vs control .12), and waist circumference (intervention1.8 cm vs. control 2.8 cm per year of age).

**Table 5:** Multilevel regression analysis for body composition variables

	Estimated % body fat			BMI			BMI-SDS			Waist circumference		
	β	CI	p	β	CI	p	β	CI	p	β	CI	p
Constant	17.7	16.9, 18.5	.000	17.0	16.6, 17.4	.000	0.3	0.1, 0.5	.000	59.7	58.5, 60.9	.000
Sex	8.2	7.5, 8.8	.000	0.4	0.1,0.7	.003	-0.1	-0.2, 0.01	.036	-1.3	-2.0, -0.5	.000
Group	1.6	0.5, 2.6	.001	1.2	0.6, 1.7	.000	0.4	0.2, 0.7	.000	2.6	1.0, 4.2	.001
Age	1.3	0.9, 1.7	.000	0.6	0.4, 0.8	.000	0.03	-0.1, 0.1	.227	2.4	1.9, 2.9	.000
Age^2^	0.5	-.04, 1.0	.035	0.3	.01, 0.5	.019	0.1	-.01, 0.2	.036	0.4	-0.2, 1.0	.102
Constant	-0.9	-1.7, -0.2	.005	-0.4	-0.8, -0.1	.003	-0.2	-0.3, -0.1	.002	-1.0	-0.2, -2.5	.007

### Knowledge of healthy lifestyles

The results for knowledge of healthy lifestyles are shown in Table 4. At baseline there was no difference between boys and girls or between groups in their knowledge of healthy lifestyles. There was a small but significant increase in knowledge across the intervention period (.08 marks for every month, so about 1 mark or 10% in total) in the control group but no significant group by time interaction.

### Psychological measures

The results for the psychological measures are shown in Table 6. Few significant differences were found in these variables and only extrinsic motivation showed a group by time interaction, with extrinsic motivation decreasing more in the intervention group compared to the control group.

**Table 6:** Multilevel regression analysis for psychological variables

	Enjoyment of physical activity			Perceived competence			Intrinsic motivation			Extrinsic motivation		
	β	CI	P	β	CI	p	β	CI	p	β	CI	p
Constant	18.9	18.6, 19.2	.000	15.5	14.9, 16.2	.000	4.7	4.6, 4.8	.000	2.4	2.2, 2.6	.000
Sex	0.0	-0.2, 0.2	.345	-0.8	-1.1, -0.5	.000	-0.01	-0.1, 0.0	.309	-0.1	-0.2, 0.0	.023
Group	0.5	0.1, 1.0	.009	0.5	-0.2, 1.3	.09	0.1	0.1, 0.2	.001	0.2	-0.1, 0.5	.115
Months in intervention	0.0	-0.03, 0.1	.309	0.0	-0.1, 0.0	.092	0.01	0.0, 0.02	.006	-0.04	-0.1,-0.02	.000
Group × month	0.0	-0.1, .01	.067	-0.1	-0.1, 0.0	.106	-0.01	-0.03, 0.01	.159	-0.02	-.04, -0.0	.023

### Feedback from teachers and parents

Classroom teachers and head-teachers were interviewed at the midpoint and the end of the intervention to establish their views of the programme, how they had used it, and how it could be improved. Two parents from each class at each intervention school (n = 24) were interviewed over the telephone to establish their views of the programme and its impact. Teachers reported using the intervention resources in a variety of ways including as a special topic over one week focused on healthy lifestyles and as a weekly topic. Teachers also valued the cross-curricular nature of the resources. For example, one teacher reported that "we used some of the CD. It had mathematical, it had cross-curricular activities where the children did exercises like pie charts of what they ate and whether they walked to school, what activities they did throughout the day. Also about the heart and about biology". For the majority of teachers the main focus had been working towards the one mile runs/walks and teachers reported that the highlight runs had provided a very important goal for the children in encouraging them to increase their physical activity. For example one teacher commented that the highlight runs were "massive, I think because it gives them something to aim for...as well as just doing it for health and fun, they see an end product." Teachers also commented on the importance of strong head-teacher support to the success of the intervention as the following quote illustrates: "you will probably find that the schools that you go into with the greatest success stories are the ones where the principal is totally committed to it and is making it high profile". It was also felt that the intervention was most successful when it was taken on board as a whole school initiative with staff providing organised activities for the children. Teachers also considered it important that all adults within the school (including for example dinner ladies and support staff) were involved so that the children see all of the adults in the school are supporting healthy living. For future interventions teachers felt that: (a) a different highlight event should be included in each term for each year so that when children progressed from one year to the next there were different goals to strive for. For example one teacher commented that highlight events should "*[change] *each term but also you could have a different activity for each year group, so that when they change they can look forward to that activity and try to do better than the class last year"; (b) inter-class competitions with prizes for the amount of physical activity undertaken would be very helpful; and (c) testing and measurements should be part of future interventions as the pedometers were very popular with the children and highlighted to them how active or inactive they were. Parents felt that wearing pedometers helped their children to become more active and that their children were more aware of why they should be more active, how much activity they were undertaking and why they should consume more fruit and vegetables.

## Discussion

This school-based intervention in 7–11 year-old children resulted in significant increases in physical activity, suppression of the age-related rate of increase in body composition variables, and reductions in extrinsic motivation. There was no intervention effect on fruit and vegetable consumption, other psychological variables, and distance run in the multi-stage shuttle run.

The increases in MVPA and steps observed in the current study are in contrast to previous primary school based interventions in the UK [8,17,18] which reported no changes in physical activity. At least two possible explanations exist for these differences; (i) the current intervention content was more effective; or (ii) the greater sensitivity of the objective measures of physical activity employed in the current study enabled small but significant changes to be observed. Systematic reviews have reported that intervention studies using objective measures are more likely to report significant positive results than studies employing a self-reported measure [20,21]. It is encouraging that the changes in physical activity were also observed in the least active participants at baseline, demonstrating that the current intervention has broad appeal and reaches the group most at risk from physical inactivity.

The current results support the conclusion of Flodmark [23] that it is possible for school-based interventions to impact positively on body composition. However, the body composition outcomes in studies within Flodmark's review were not always associated with changes in physical activity levels (e.g., the changes may have been associated with changes in diet and/or sedentary behaviour). Likewise, changes in body composition may have been associated with changes in multiple-risk factors. For example, Mueller et al [16], reported a lower increase in skinfold thickness in the intervention group over a 3 month period, however these changes were associated with increases in physical activity, fruit and vegetable intake and intake of food with low fat content and decreases in television viewing. The current findings suggest that increases in physical activity alone may be effective in facilitating changes in body composition. In a cross-sectional study it was reported that increases of 15 minutes per day in MVPA were associated with lower odds of obesity in boys and girls [44]. Our results demonstrate that even with smaller increases in daily MVPA there may be positive effects on body composition in children.

The lack of change in fruit and vegetable consumption is disappointing. There are several possible explanations for this, and it is likely that the full explanation involves an element of all of them. The results may reflect that dietary behaviour is more difficult to change because of the gate-keeping role of parents and others in the provision of food and thus targeting children alone is likely to be insufficient to facilitate change [45]. It may also reflect the difficulties in accurately assessing diet in this age group in general and more specifically the fact that the 24 hour recall employed could not accurately assess quantities [17]. Alternatively, it may be that the nutrition content within the intervention was not employed to the same degree by teachers or that the strategies were less extensive, and therefore less effective, than those for physical activity (for example, there was not a corresponding festival event for nutrition and the holiday wall planner focussed on physical activity). Supporting this argument, Doak et al [22] have argued that how an intervention addresses a behaviour change is crucial and that the level of active engagement by participants may influence outcomes. In the current intervention, the nutrition component was an education-based intervention teaching the children about healthy foods and encouraging them to make healthy choices, whereas the physical activity component went beyond this educative approach by including opportunities to be active and providing festival events to work towards.

The strengths of this intervention lie in the delivery of the intervention by school personnel, its fit with the demands of the national curriculum, and its flexible design which respected the autonomy of teachers by allowing them to decide how and when they would use the intervention material. These features improve the potential for the future sustainability of the intervention. In addition, the cross-curricula links allow for a consistent message in a range of subjects [8,46,47].

An important strength of the current research is the use of objective measures of physical activity which provide a rigorous and sensitive test of the intervention effects and removes the bias associated with self-report. Furthermore, we employed multiple measures of body composition rather than relying on BMI. While BMI is acceptable and practical in large trials it is not ideal as it does not distinguish between changes in muscle, bone and fat [39]. Use of skinfold measurements allows for predictions of body fat percentage and therefore provides a better indication of changes in adiposity than BMI. In addition, waist circumference has been advocated as a more sensitive index of obesity prevalence in young people than the BMI [48]. Studies with multiple measures have the advantage of being able to provide a clearer picture of the intervention effect on body composition [22] especially when increasing physical activity is a goal of the intervention. For example, increases in physical activity could lead to a reduction in skinfolds but no changes in BMI or BMI centile as the physical activity may maintain or even increase the child's lean body mass.

Limitations of this study are: (i) due to the local media content it was not possible to conduct a randomised control trial. However, we did match the schools on key variables and there is debate as to the appropriateness of RCTs for evaluating health promotion interventions [49,50]; (ii) assessment of diet may not have been sensitive enough; (iii) the lack of a long term follow-up as yet means the sustainability of behavioural changes can not be assessed; (iv) while we attempted to match the groups on SES this was not successful and group level matching was not reflected at the individual level resulting in the intervention group being of lower socioeconomic status than the control.

Questions remain as to how to effect favourable changes in diet through school based interventions. Greater links with families are most likely required but the exact nature and contribution of this involvement remains unclear [22]. The highlight events were a key feature of the intervention but it is unclear how often the content of these events would need to change to maintain interest.

This systematic evaluation shows that objectively measured physical activity and body composition can be successfully targeted in primary schools with relatively few additional resources. Given the positive effects achieved we believe the programme could be successful in other primary schools.

## Competing interests

The authors declare that they have no competing interests.

## Authors' contributions

TG, participated in the design of the study, managed the data collection process and drafted the manuscript. MN, JG, and DS participated in the design of the study, the data collection process and helped to draft the manuscript. AN performed the statistical analysis. All authors read and approved the final manuscript.
